# Potential Effects of Phytoestrogen Genistein in Modulating Acute Methotrexate Chemotherapy-Induced Osteoclastogenesis and Bone Damage in Rats

**DOI:** 10.3390/ijms160818293

**Published:** 2015-08-06

**Authors:** Tristan J. King, Tetyana Shandala, Alice M. Lee, Bruce K. Foster, Ke-Ming Chen, Peter R. Howe, Cory J. Xian

**Affiliations:** 1 Sansom Institute for Health Research, School of Pharmacy and Medical Science, University of South Australia, Adelaide, SA 5001, Australia; E-Mails: tristan.king@unisa.edu.au (T.J.K.); tetyanas@geneworks.com.au (T.S.); alice.lee@unisa.edu.au (A.M.L.); 2 Department of Physiology, School of Medical Sciences, University of Adelaide, Adelaide, SA 5001, Australia; 3 Department of Orthopaedic Surgery, Women’s and Children’s Hospital, North Adelaide, SA 5006, Australia; E-Mail: Bruce.Foster@health.sa.gov.au; 4 Institute of Orthopaedics, Lanzhou General Hospital, Lanzhou Command of Chinese People’s Liberation Army, Lanzhou 730050, China; E-Mail: chenkm@lut.cn; 5 Nutritional Physiology Research Centre, School of Health Sciences, University of South Australia, Adelaide, SA 5001, Australia; E-Mail: peter.howe@newcastle.edu.au; 6 Clinical Nutrition Research Centre, University of Newcastle, Callaghan, NSW 2308, Australia

**Keywords:** methotrexate, chemotherapy, osteoporosis, genistein

## Abstract

Chemotherapy-induced bone damage is a frequent side effect which causes diminished bone mineral density and fracture in childhood cancer sufferers and survivors. The intensified use of anti-metabolite methotrexate (MTX) and other cytotoxic drugs has led to the need for a mechanistic understanding of chemotherapy-induced bone loss and for the development of protective treatments. Using a young rat MTX-induced bone loss model, we investigated potential bone protective effects of phytoestrogen genistein. Oral gavages of genistein (20 mg/kg) were administered daily, for seven days before, five days during, and three days after five once-daily injections (sc) of MTX (0.75 mg/kg). MTX treatment reduced body weight gain and tibial metaphyseal trabecular bone volume (*p* < 0.001), increased osteoclast density on the trabecular bone surface (*p* < 0.05), and increased the bone marrow adipocyte number in lower metaphyseal bone (*p* < 0.001). Genistein supplementation preserved body weight gain (*p* < 0.05) and inhibited *ex vivo* osteoclast formation of bone marrow cells from MTX-treated rats (*p* < 0.001). However, MTX-induced changes in bone volume, trabecular architecture, metaphyseal mRNA expression of pro-osteoclastogenic cytokines, and marrow adiposity were not significantly affected by the co-administration of genistein. This study suggests that genistein may suppress MTX-induced osteoclastogenesis; however, further studies are required to examine its potential in protecting against MTX chemotherapy-induced bone damage.

## 1. Introduction

Chemotherapy is known to cause significant adverse effects in the bone, notably bone growth arrest and/or diminished bone mineral density (BMD) in growing or adult bones [1,2,3,4]. Whilst there is evidence to suggest some changes may be acute, the use of chemotherapy to treat childhood malignancies is strongly associated with a persisting low BMD long after the cessation of treatment [4,5]. Chemotherapy of childhood cancers is known to negatively impact a crucial window of bone growth and bone mass accrual [6]. As a result, failure to reach an optimal peak BMD during puberty can lead to a lower-than-average adult BMD, an outcome linked to an increased risk of fracture [1,7,8,9,10,11].

Methotrexate (MTX) is an anti-metabolite chemotherapeutic which inhibits the utilisation of folate during *de novo* purine and thymidine synthesis, in turn disrupting DNA/RNA synthesis and cell replication [12]. Therapeutically, MTX is a common choice for the treatment of childhood cancers such as acute lymphoblastic leukaemia (ALL) and, consistent with other chemotherapeutics, has been shown to induce bone growth arrest and diminished BMD in these patients [2,10,13,14,15] as well as in long-term follow up in survivors [16,17]. As a result of its common use, treatment success, and associated bone-related side effects, MTX has become an increasingly studied chemotherapeutic in relation to understanding the underlying mechanisms of and preventing the associated bone defects.

Some previous studies have shown a link between MTX-induced bone loss and alterations to the bone remodeling balance and environment following MTX administration. Altered levels of serum bone turnover markers in ALL patients receiving MTX indicated increased bone resorption (carried out by osteoclasts) and, conversely, diminished bone synthesis (carried out by osteoblasts) [13,15]. Consistently, these effects are reflected in experimental studies examining the effect of MTX in long bones of rodents [18,19]. Supportively, the administration of MTX acutely affects both the densities of the remodeling cells (diminished osteoblast numbers but increased osteoclast presence on trabecular bone surfaces) [18,20,21,22]. More recently, this has been shown to be related to the altered differentiation potential of the bone cells from their respective precursors within the bone marrow. A switch in lineage determination from the remaining common bone marrow stromal precursor cells has been observed, favouring adipocyte formation rather than the formation of osteoblasts, a change which leads to the increased presence of fat (adiposity) within bone marrow [23,24]. In addition, increased osteoclast formation from the bone marrow cells following MTX administration has been linked to the onset of a local and systemic pro-inflammatory environment with the elevated expression of pro-osteoclastogenic cytokines, which are capable of inducing osteoclast formation both *in vivo* and *ex vivo* [25]. Thus, MTX can induce a reduction in bone volume via disruption to normal bone remodeling with reduced bone formation but increased resorption.

Currently, there is no accepted specific supplementary treatment aimed at reducing or preventing MTX chemotherapy-induced bone damage. Traditional anti-resorptive therapies such as the bisphosphonates (Alendronate) and receptor activator of NF-κB ligand (RANKL) antibodies (Denosumab) may offer bone protective effects against cancellous and spongy bone loss caused by the malignancy and/or therapies, including hormone/androgen therapy (HRT), radiotherapy, and chemotherapy [26]. However, whilst they are the gold standard for osteoporosis management, their use for preventing cancer or therapy-induced bone loss is associated with significant complications such as osteonecrosis of the jaw and/or disruption of normal bone remodeling [27,28]. In this respect, alternative therapies devoid of significant side effects or contraindications may be appealing.

Currently, there have been some recent rodent experimental studies examining potential benefits of natural substances in attenuating MTX adverse side effects, including those of folinic acid (folate analogue) [29] and fish oil [30]. Recent studies have also shown potential benefits of genistein, a phytoestrogen with known bone-sparing properties. Whilst there is some conjecture about the bone protective effects of genistein within post-menopausal women [31], there is a considerable amount of evidence for its benefit [32]. One of the first observations of the potential bone protective effect of genistein was observed in Asian populations of post-menopausal women where it was reported that those who consume diets rich in soy suffered fewer fractures than Caucasian women [32], an outcome attributed to an increase in BMD [33,34]. Consistently, genistein has demonstrated positive anabolic effects upon BMD in ovariectomised (OVX) rodents where its supplementation promotes bone formation and inhibits the bone loss associated with estrogen depletion [35,36,37]. Genistein has been shown to be able to promote osteogenesis and inhibit osteoclastogenesis, thus positively affecting the bone remodeling both *in vitro* and *in vivo* [38].

With our improved understanding of the mechanisms associated with MTX-induced bone loss, the current study investigated the effects of genistein supplementation upon MTX-induced changes in bone volume and the bone remodeling cells with tibial metaphysis (the region with the most active bone remodeling) at a critical time-point (day nine following the first of five consecutive once-daily MTX injections) when pronounced bone damage is known to occur [22,25].

## 2. Results

### 2.1. Effects on Body Weight Changes

**Figure 1:**
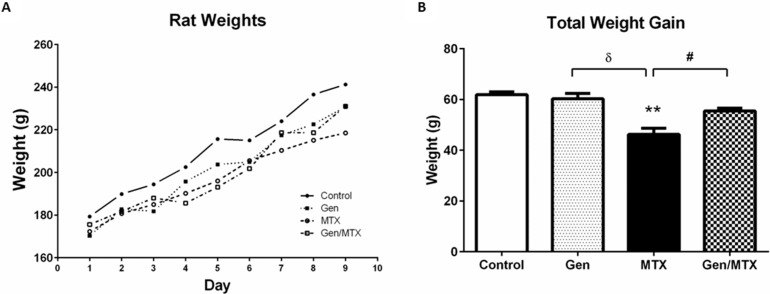
Effects of methotrexate (MTX) with/or without genistein on body weight gain. (**A**) Representative graph of weight accumulation over the period of treatment illustrating that MTX treatment (open circles, dashed line) slowed body weight gain, whilst the MTX and genistein combination (Gen/MTX, open square, dashed line) recovered their weight by the end of the trial; (**B**) Total body weight gained over the period of MTX administration until specimen collection. MTX significantly reduced weight accrual when compared to control group (** *p* < 0.01) or to the Gen alone group (^δ^ *p* < 0.001), and Gen supplementation (Gen/MTX) significantly attenuated the weight accrual suppression when compared to MTX alone group (^#^ *p* < 0.05) (*n* = 6).

Consistent with previous studies, five daily injections of MTX caused considerable attenuation of body weight gain (Figure 1) with MTX-treated rats gaining, on average, 46.2 ± 2.5 g whilst control rats gained 61.9 ± 1.1 g from day one (D1) to D9 (*p* < 0.01) (Figure 1B). Overall, genistein oral gavage (15 consecutive days, including during the five days of MTX administration) was well-tolerated with no observable changes in appearance or behavior of the rats in the genistein alone group. Whilst the genistein plus MTX group (55.4 ± 1.2 g from D1 to D9) did not achieve the same total weight gain as saline control rats (*p* > 0.05), the weight increase was significantly greater than in those treated with MTX alone (*p* < 0.05) (Figure 1B).

### 2.2. Effects on Bone Volume and Trabecular Structure and Bone Turnover

**Figure 2:**
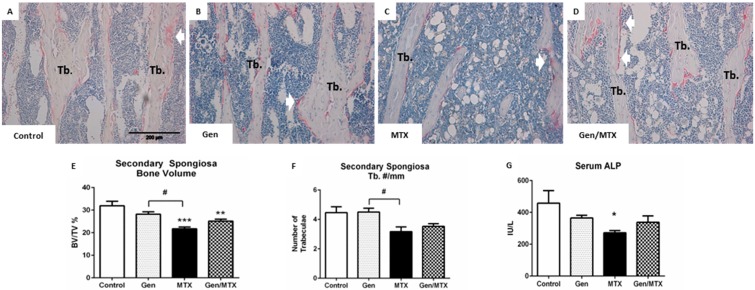
MTX reduced metaphyseal trabecular bone volume and serum levels of alkaline phosphatase (ALP) compared to the control. (**A**–**D**) Representative images (20×) of haematoxylin and eosin (H&E) and tartrate-resistant alkaline phosphatase (TRAP, a marker for osteoclasts)-stained sections for control, MTX-, genistein (Gen)-, and Gen/MTX-treated groups, respectively. Pink staining indicates osteoclasts (white arrows) which line the trabecular bone (Tb) surface; (**E**) Quantification of bone volume measured as the percentage of total tissue area occupied by trabecular bone (BV/TV%); (**F**) Quantification of the number of trabeculae spanning the width of the secondary spongiosa (Tb.#/mm); (**G**) Serum analysis of ALP levels. * *p* < 0.05, ** *p* < 0.01 and *** *p* < 0.001 *vs.* control; ^#^ represents a statistical difference between treatment groups with *p* < 0.01 (*n* = 6).

Effects of MTX and/or genistein treatments upon tibial bone histomorphometry were assessed in the secondary spongiosa of the tibial metaphysis (Figure 2), a region shown previously to have bone loss after MTX treatment [22]. Trabecular bone volume (BV/TV%) was significantly reduced in MTX alone rats (*p* < 0.001) and in MTX plus genistein-treated rats (*p* < 0.01) when compared to saline controls (Figure 2A,B *vs.* Figure 2C,E). No difference was observed between control and genistein alone rats (Figure 2A *vs*. Figure 2B,E). The reduction in bone volume was not as severe in the case of MTX co-administered with genistein, although it was still significantly less than the control (*p* < 0.01). The mean bone volume for controls was 31.91%, whilst the mean value of the MTX and genistein combination (Gen/MTX) rats was higher than that of MTX treated rats, 25.16% *vs.* 21.71%, respectively, and the effect of combined treatment did not result in a significant difference between the latter two groups (*p* = 0.1405).

As trabecular bone mass is related to bone architecture, the trabecular number, thickness, and spacing were also examined. No significant differences were observed between any groups with regards to spacing and thickness. However, compared to the genistein alone group, MTX treatment significantly reduced the number of trabeculae in the metaphysis (*p* < 0.01) (Figure 2B *vs.* Figure 2C,F). Co-treatment of MTX with genistein was not significantly different from MTX alone in regard to affecting trabecular number, thickness, or spacing.

As an assessment of the treatment impact on bone formation, the serum concentrations of bone formation marker alkaline phosphatase (ALP) were measured and were shown to be significantly reduced following MTX treatment when compared to the control (Figure 2G). However, there were no differences between other groups, and genistein in combination with MTX did not rescue this MTX effect.

### 2.3. Effects on Densities of Bone Modelling/Remodeling Cells within the Metaphysis

**Figure 3:**
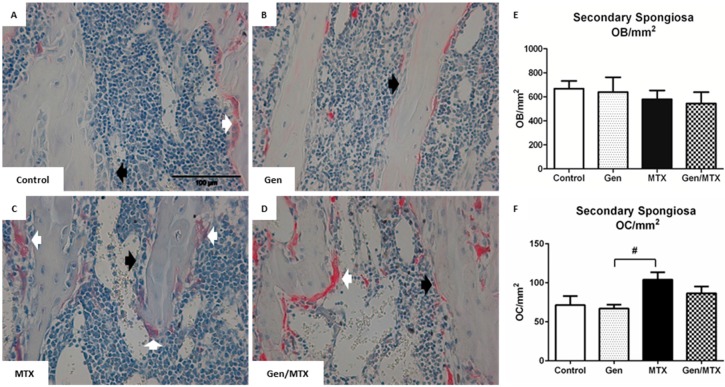
MTX treatment increased the density of bone-resorbing osteoclasts and genistein had no significant effect on densities of the remodeling cells. (**A**–**D**) Representative images (40×) of H&E-stained tibial metaphysis sections from a control, genistein (Gen)-, MTX-, or genistein + MTX (Gen/MTX)-treated rat, respectively, which have been counterstained with TRAP for osteoclast identification (white arrows: osteoclasts or OC; black arrows: osteoblasts or OB); (**E**) Quantification of osteoblast density (OB/mm^2^) within the secondary spongiosa region of the metaphysis; (**F**) Quantification of osteoclast density (OC/mm^2^) identified as multinucleated (≥3 nuclei) TRAP^+^ cells (white arrows). *n* = 6, ^#^ *p* < 0.05 between MTX and Gen groups.

To assess if genistein had positive effects on bone modelling that may protect against MTX-induced bone damage, the respective densities of osteoblasts and osteoclasts within the metaphysis were assessed (Figure 3). The density of osteoblasts (black arrows) was consistent for all treatment groups (Figure 3A–E). However, the numbers of osteoclasts, identified as large, tartrate-resistant alkaline phosphatase (TRAP)^+^, and multinucleated cells lining the trabecular surface (white arrows Figure 3A–D), were greatest in MTX-treated rats, and these were significantly greater than those treated with genistein alone (*p* < 0.05) (Figure 3B,F *vs.* Figure 3C). Co-treatment (Gen/MTX) did not significantly attenuate this effect despite the small trend to yield fewer osteoclasts/mm^2^ than MTX alone (86.9 ± 8.7 and 104 ± 9.4, respectively; *p* = 0.43). Noticeably, MTX treatment did not significantly increase the number of osteoclasts lining the trabecular surface when compared to control animals (*p* = 0.104).

### 2.4. Effects on ex Vivo Osteoclastogenesis

**Figure 4:**
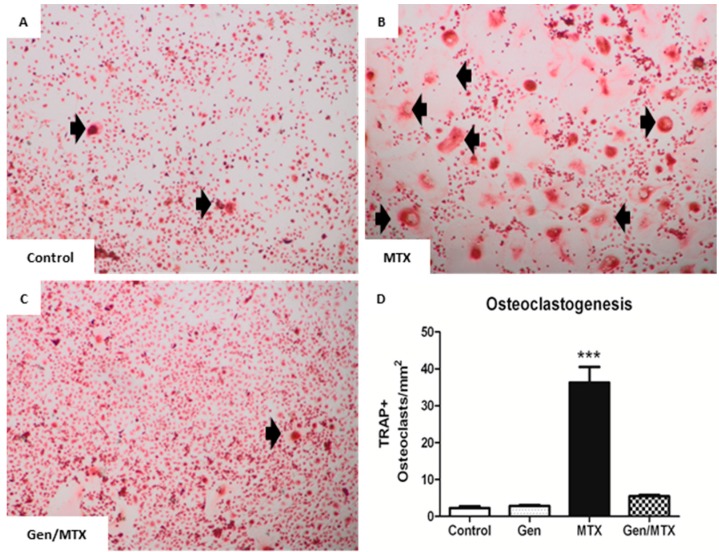
Co-treatment with genistein (Gen) significantly suppressed MTX-induced *ex vivo* osteoclast formation. (**A**–**C**) Representative images (20×) of osteoclasts differentiated in the presence of macrophage colony stimulating factor (M-CSF) and receptor activator of NF-κB ligand (RANKL) from bone marrow mononuclear cells isolated from control, MTX-, and Gen + MTX-treated rats, respectively. Cells were subsequently stained with TRAP to identify osteoclasts; (**D**) Osteoclasts were identified as large, TRAP^+^, and multinucleated cells (≥3 nuclei) (black arrows in panels **A**–**C**) and quantified as osteoclasts/mm^2^ of culture dish. *n* = 6, *** *p* < 0.001 *vs.* control.

Genistein is known to reduce bone resorption *in vivo* [39] and also suppress osteoclast formation *in vitro* [37,40]. Here, the effect of genistein treatment *in vivo* upon the potential of the bone marrow cells of treated rats to form mature osteoclasts *ex vivo* was examined in macrophage colony stimulating factor (M-CSF) and RANKL-induced osteoclastogenesis cultures (Figure 4). Osteoclasts (black arrows), identified via light microscopy as large TRAP^+^ multinucleated (≥3 nuclei) cells, were apparent in cultures from all treatment groups (Figure 4A–C) but were present in significantly greater numbers in cultures of MTX alone-treated rats (*p* < 0.001) (Figure 4A *vs.* Figure 4B,D). Noticeably, the co-treatment of MTX with genistein significantly suppressed the effect of MTX-induced osteoclastogenesis (5.5 ± 0.4 *vs.* 36.3 ± 4.2 osteoclasts/mm^2^, respectively, *p* < 0.001 between the MTX plus genistein group and the MTX alone group) (Figure 4C,D).

### 2.5. Effects on Expression of Osteoclastogenesis-Related Genes in Metaphyseal Bone

**Figure 5:**
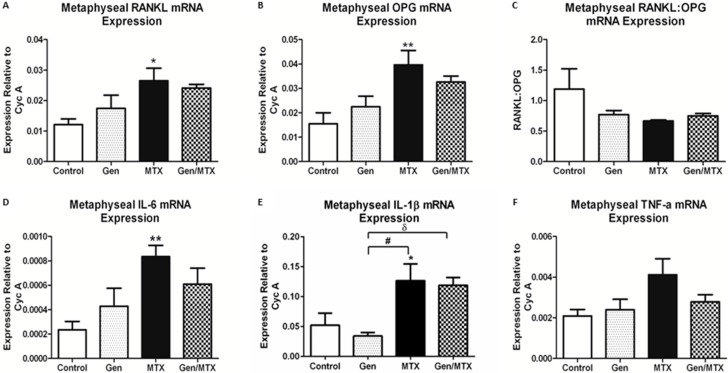
MTX administration increased mRNA expression of receptor activator of NF-κB ligand (RANKL), osteoprotogerin (OPG), interleukin-6 (IL-6), and IL-1β in the metaphysis bone compared to control rats. (**A**,**B**) Quantitative RT-PCR of osteoclastogenesis regulators RANKL and OPG, respectively, within the metaphysis; (**C**) Ratio of relative mRNA expression of RANKL to OPG; (**D**–**F**) Quantitative RT-PCR of pro-inflammatory cytokines IL-6, IL-1β, and tumor necrosis factor-alpha (TNF-α), respectively. *n* = 6, * *p* < 0.05 and ** *p* < 0.01 *vs.* control; ^#^ *p* < 0.01 between Gen and MTX alone groups, and ^δ^ *p* < 0.05 between Gen alone and Gen/MTX groups.

To examine if genistein offered protection against MTX-induced damage by modulating the expression of factors regulating osteoclast formation and activity, the mRNA expression in metaphysis bone was analysed for osteoclastogenesis regulatory factors, including RANKL and osteoprotogerin (OPG) as well as pro-inflammatory cytokines including tumor necrosis factor-alpha (TNF-α), interleukin-1 (IL-1), and IL-6. Following MTX administration, both the critical osteoclastogenic factor RANKL and inhibitor OPG (RANKL decoy receptor) demonstrated significant elevation in mRNA expression (*p* < 0.05 and *p* < 0.01 *vs.* control); however, their expression was not affected by other treatments (Figure 5A,B). The ratio of expression of RANKL to OPG (RANKL:OPG) was unaffected by any treatments, despite the obvious changes in individual expression levels (Figure 5C). Since IL-6, IL-1β, and TNF-α are known to augment osteoclast formation and activity, their relative expression levels were also analysed in the metaphysis bone. MTX alone induced an increase in IL-6 (*p* < 0.01 *vs.* control) and IL-1β (*p* < 0.05) expression (Figure 5D,E) and tended to increase TNF-α (*p* = 0.0553) expression (Figure 5E). Noticeably, both MTX alone and MTX plus genistein groups displayed greater expression of IL-1β when compared to the genistein alone group (*p* < 0.05) (Figure 5E).

### 2.6. Bone Marrow Adiposity

**Figure 6:**
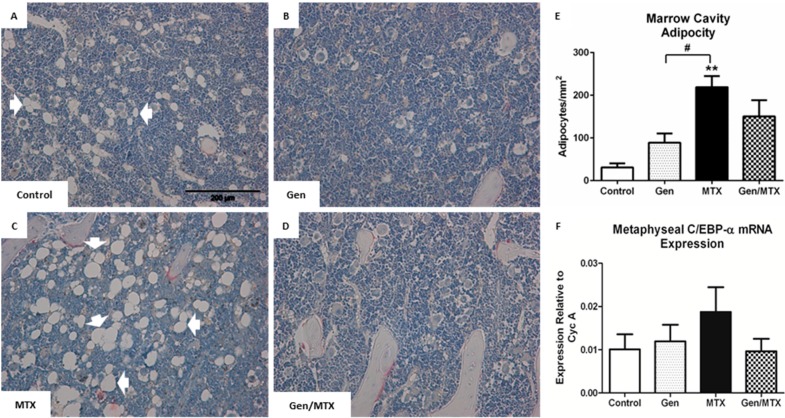
Changes in adipocyte density within the lower secondary spongiosa of the tibia in rats following various treatments. (**A**–**D**) Representative photographs (20×) of sections stained with H&E for control, genistein (Gen), MTX, and Gen + MTX groups, respectively. Adipocytes (white arrows) are easily identified as empty spaces within the marrow cavity which are encased by a membrane; (**E**) The density of adipocytes was quantified as the number of adipocytes/mm^2^ of marrow cavity excluding bone area; (**F**) Adipogenesis transcription factor CCAAT/enhanced binding protein-α (C/EBPα) relative mRNA expression as quantified via RT-PCR. *n* = 6, ** *p* < 0.01 *vs.* control, and ^#^ *p* < 0.05 between Gen and MTX groups.

Adipocytes, which are found interspersed within marrow cells in the lower metaphysis and diaphysis of long bones, have been shown to be sensitive to MTX chemotherapy and thus were examined here (Figure 6). MTX significantly increased the density of adipocytes within the marrow cavity of the lower metaphysis when compared to the control (*p* < 0.01) and genistein alone (*p* < 0.05) groups (Figure 6A,B *vs.* Figure 6C,E). Whilst the bones of rats with the combination treatment of genistein with MTX (Gen/MTX) displayed fewer adipocytes, this was not significantly lower than those arising from MTX alone (Figure 6C *vs.* Figure 6D,E). Since an increase in the expression of adipogenic transcription factor CCAAT/enhanced binding protein-α (C/EBPα) mRNA expression is associated with initiation of adipocyte formation, its relative expression level within the metaphysis was also examined to see if genistein affected adipogenesis following MTX treatment. MTX alone-treated animals tended to exhibit a higher expression level compared to saline controls (control *vs.* MTX, *p* = 0.9064), whilst the Gen/MTX combination treatment tended to attenuate MTX-induced C/EBP-α expression (MTX *vs.* Gen/MTX, *p* = 0.9275) (Figure 6F).

## 3. Discussion

The use of MTX as a chemotherapeutic commonly causes bone-related defects including bone growth arrest, disruption of bone remodeling, diminished BMD, and an increased likelihood of fracture. Despite these significant and ongoing side effects, there are currently no specific therapies employed in an effort to prevent or attenuate their onset. Based on our understanding of the effect of MTX upon the bone remodeling cells, and the mechanisms through which this occurs, genistein became a compound of interest for therapeutic exploration. In a number of studies *in vivo*, genistein has been reported to promote bone mass preservation via an increase in bone formation and a decrease in bone resorption [33,34,41,42]. This current study demonstrates that genistein had minimal protective effect upon MTX-induced damage in bone volume and structure, densities of osteoblasts and osteoclasts, and bone marrow adiposity. However, genistein did afford significant protection against MTX-induced osteoclastogenesis, which is a prominent mechanism mediating MTX-induced bone loss [25].

As this is one of the first cases of genistein being tested as an adjuvant therapy during MTX administration, we selected the dose of genistein at 20 mg/kg based on those shown to have positive effects in OVX bone loss models (10–45 mg/kg/day) [35,36,37]. Higher doses of up to 500 mg/kg/day have been associated with osteopetrosis and alteration to the gonadal function in both females and males [43]. Of note, our chosen dose of 20 mg/kg/day is well below the minimum adverse effect level associated with these negative changes [43], and we found the administration of genistein at this dose to be well tolerated in rats. However, the treatment regimen of the current study is a potential limitation where a singular daily oral gavage may not be effective in providing beneficial effects in bone, as the serum levels of genistein achieved via this delivery may not be sufficient to cause significant effects and those levels may not reflect those achievable as a dietary supplement [44]. Nevertheless, the administration of genistein with this dosing regimen appeared to have improved the health of rats during the administration of MTX as observed from the rats’ general well-being, and the extents of MTX-induced gastrointestinal side effects and bone weight gain during the treatment period. Previously, we have noted that rats receiving chemotherapeutic doses of MTX suffer from gastrointestinal irritation and malnourishment [45,46], an effect evident in MTX alone-treated rats in this study. Using weight gain as a crude indicator of nutritional health and overall growth, we found that MTX-treated rats supplemented with genistein regained weight quicker than their MTX alone counterparts and achieved a similar total weight gain as control rats by the end of the study. Collectively, this study suggests that genistein may help to relieve or aid in the repair of gut mucosal irritation caused by MTX, and that it may be a source of secondary benefit on bone health, considering that nutrition and mobility play significant roles in bone mass accrual and maintenance, especially during this period of bone development.

Consistent with our previous studies [22,25], MTX caused a reduction in the total bone volume measured within the secondary spongiosa; however, whilst this is usually associated with altered bone architecture, in this case MTX did not alter the number, thickness, or spacing of trabeculae. Genistein treatment alone, which has been shown to alter bone mass and architecture in some bone loss model studies [36,47,48], did not affect the bone volume or number of trabeculae within the secondary spongiosa in saline control-treated normal rats. Similarly, when given in combination with MTX, it did not significantly attenuate MTX-induced changes. Overall, the effect on bone morphometry of genistein at this dose, in combination with MTX treatment, is difficult to conclude in this particular study using this acute treatment setting. In order to truly assess genistein’s potential during MTX chemotherapy, more studies are required with both acute and long-term MTX treatment models with different doses.

Typically, negative changes in the densities of the bone remodeling cells within the metaphysis accompany a decrease in the bone volume; hence, the suitability of genistein in preventing this was assessed here. Consistently, with bone morphology in this study, MTX did not significantly alter the densities of the bone remodeling cells although MTX treatment appeared to increase osteoclast density on bone surfaces. Consistent with genistein’s effects upon bone volume and architecture, treatment with genistein alone or in combination with MTX did not cause significant changes in osteoclast or osteoblast densities. Interestingly, whilst the genistein alone group was not different to controls in osteoclast density, there were significantly fewer osteoclasts in the genistein alone group compared to the MTX alone group.

A recent development in the understanding of MTX chemotherapy-induced bone loss has highlighted the role of osteoclasts in this process. It was shown that an increase in osteoclast density within the metaphysis is associated with an increase in the bone marrow's potential to form osteoclasts [22,25]. As expected, MTX treatment increased the osteoclastogenic potential of the bone marrow precursor cells *ex vivo*. Previously, this MTX-induced osteoclastogenesis has been linked with changes in osteoclastogenic cytokine gene expression within the metaphysis as well as with an increase in serum concentration [25], an observation that was also seen in patients treated with chemotherapy as well as *in vitro* [49,50]. In the current study, MTX increased the mRNA expression for IL-1β and IL-6 and also appeared to induce TNF-α (*p* = 0.0552) despite the lack of effect in modulating the RANKL/OPG expression ratio. In this study, genistein’s most notable protective effect against MTX-induced changes within the bone was found to center on an ability to significantly inhibit the MTX-induced increase in bone marrow osteoclastogenic potential. Although genistein supplementation displayed trends of suppressing MTX-induced cytokine expression, statistically, none of these changes are significant. However, the MTX/genistein combination treatment was demonstrated to have a significant and dramatic effect upon *ex vivo* osteoclast formation from bone marrow cells of treated rats. This suppressing effect *ex vivo* appears to be consistent with the effect of the combination treatment in non-significantly lowering the MTX-induced osteoclast density on bone surface *in vivo*. Previously, genistein has been shown to attenuate osteoclast formation and activity *in vitro* [40,51], and genistein has been reported to inhibit inflammation-induced osteoclast formation and activity and bone loss through modulating the expression of pro-osteoclastogenic cytokines TNF-α, IL-1, and IL-6 [52,53]. Further studies will be required to study the potential effects and action mechanisms of genistein in modulating osteoclast formation and activity in cancer chemotherapy bone loss settings.

Apart from analyzing treatment effects on osteoclastogenesis and the presence of bone-resorbing osteoclasts, the current study has also examined treatment effects on osteoblast density and bone formation. We found that whilst MTX treatment did not significantly decrease osteoblast density (data not shown), it caused a reduction in bone formation as assessed by serum ALP levels. This observation is consistent with previous findings that MTX reduces the osteogenic potential of bone marrow stromal progenitor cells [23,24]. We also found that whilst genistein supplementation did not significantly prevent this decrease in ALP levels, it had a moderate but statistically non-significant effect in attenuating a MTX-induced decrease in this bone formation marker. Genistein is known to have pro-osteoblastic effects *in vitro* and osteoblasts treated with genistein increase their rate of osteogenesis/mineralization and the expression of bone protein osteocalcin [54,55]. Consistently, genistein treatment delayed or prevented bone loss in the OVX model of osteoporosis [56,57]. Further studies with various doses and treatment protocols are required to investigate the potential pro-osteogenic effects of genistein in MTX chemotherapy models.

In some previous studies, the MTX-induced decrease in osteogenic potential was shown to be due to changes in the lineage determination of bone marrow stromal cell precursor cells, whereby adipocytes are formed in preference over osteoblasts [23,24]. One aspect of this includes an increased expression of the early adipogenic regulator C/EBP-α in MTX-treated rat stromal cells as we also showed here. Consistent with this and other previous studies, MTX caused an increase in adipocyte numbers within the bone marrow cavity [23]. Noticeably, whilst MTX alone-treated rats had significantly more marrow adipocytes than rats receiving genistein alone, genistein did not reduce adipocyte numbers significantly in MTX-treated rats, even though the marrow cavity appeared healthier as a result. Consistently, the expression of C/EBP-α was highest in the MTX-treated animals and the co-administration of genistein seemed to attenuate this increase, though the effect was statistically non-significant. Of note, genistein at a higher dose has been demonstrated to reduce adipogenic deposition in rodents previously; importantly, whilst 20 mg/kg dose only demonstrated a trend, higher doses caused significant effects [58], suggesting that an increased genistein dose may show more significant effects in attenuating MTX-induced adiposity. Hence, it is apparent that whilst MTX has been consistently shown to cause bone marrow adiposity, further studies are required to examine the potential protective effects of genistein at different doses or treatment protocols against MTX-induced bone marrow fat accumulation.

Although some experimental studies have reported minimal effects of genistein in OVX models of osteoporosis [59,60], and a clinical study has reported evidence of post-menopausal women failing to receive benefits of genistein [31], the potential benefit of genistein supplementation in protecting bone has been demonstrated in many OVX bone loss model studies [36,39,48] and in some human dietary studies [32], and its pro-osteogenic and anti-osteoclastogenic actions have been observed in various *in vitro* and *in vivo* studies [38]. Due to its ability in positively modulate bone remodeling, the current study evaluated the potential of genistein as a bone protective agent in the setting of childhood chemotherapy-induced bone loss. In this study with this acute MTX treatment model (mimicking the acute intensive induction phase of clinical MTX treatment for the major childhood cancer acute lymphoblastic leukemia), genistein supplementation was demonstrated to have some promise in a number of areas, including protection of the general well-being and growth of the animals and, importantly, in attenuating MTX-induced osteoclast formation from the bone marrow cells. Experimentally and in the clinic, MTX treatment has been consistently demonstrated to cause bone loss due to increased bone resorption and reduced bone formation. The observed ability of genistein to suppress MTX-induced osteoclastogenesis may have significant consequences on bone health and warrants further studies. While the current study showed that genistein supplementation was not able to significantly attenuate some parameters of MTX-induced adverse effects in bone (including changes in bone volume, serum ALP level, bone marrow adiposity, and gene expression as outlined above), data from the current study has provided some information which will be useful to further investigate the potential effects and action mechanisms of genistein in protecting bone in chemotherapy-induced bone loss models, including studies with larger cohorts, across a variety of doses considering potential concerns on its usage safety [41,61], incorporating measures for monitoring its serum levels, considering potential differences of its metabolism between rodents and humans [62], and in acute and chronic treatment models (including both the acute induction phase and maintenance phase of MTX treatment) [20].

## 4. Experimental Section

### 4.1. Animal Trials with Methotrexate (MTX) and/or Genistein Administration

Male Sprague-Dawley rats aged six weeks were randomly divided into four treatment groups (*n* = 6): control, genistein, MTX, and MTX + genistein (referred to as “gen/MTX”). Over the course of the experiment, all rats were housed in standard conditions with 12 h light-dark cycles and were provided standard rat chow and water *ad lib*. All procedures were approved (project #05-08, approved on 28 March 2008) by SA Pathology Animal Ethics Committee, South Australia.

Irrespective of their allocated treatment groups, all rats received daily subcutaneous injections of MTX or saline, in combination with preventative, concurrent, and continuing oral gavages of genistein or vehicle. Injections of MTX were administered daily for five consecutive days at a dose of 0.75 mg/kg as previously described [22,25]. Genistein at 20 mg/kg or vehicle was administered once daily via oral gavage throughout the trial: for seven days prior to the first MTX injection, for five days concurrently with MTX administration, and for three days after the final MTX administration. Control groups received injections of saline and oral gavages of vehicle, MTX rats received MTX and vehicle, genistein rats received saline and genistein oral gavages, and finally the gen/MTX animals received both MTX injections and oral gavages of genistein. Oral gavages were prepared by first solubilizing genistein (Bonistein™, DSM Nutritional Products, Kaiseraugst, Switzerland) in 100% Dimethyl-sulfoxide (DMSO) at 100 mg/mL. This was further diluted in a solution of 50% Tragacanth in water to a final concentration of 5 mg/mL. The final volume of oral gavage to be administered (between 0.5 and 2.5 mL total) depended on rat body weights. Bone specimens were collected on day nine following the first MTX dosing.

### 4.2. Histological Analyses of Bone Volume, Trabecular Architecture, and Cell Densities

To investigate structural and cellular changes occurring within the metaphysis (a region of a long bone with active modelling and remodeling most affected by MTX as shown in our previous studies [22,25]), one tibia was fixed in 10% formalin for 24 h then decalcified in Immunocal solution (Decal Corp., Tallman, NY, USA) over two weeks before being halved longitudinally along the midline and processed for paraffin embedding. Sections of 4 μm thickness were obtained, mounted on Superfrost™ slides (Thermo Fisher Scientific Australia, Scoresby, VIC, Australia), and stained for tartrate-resistant alkaline phosphatase (TRAP) (a marker for osteoclasts) and haematoxylin before being examined under a light microscope. To examine treatment effects on bone architecture, bone volume as a percentage of tissue volume (BV/TV%), trabecular thickness (Tb.Thk), trabecular spacing (Tb.Spc), and trabecular number (Tb.#) were measured in the secondary spongiosa region of metaphysis bone using Bio-analysis software as we described [20,22]. In addition, cell numbers/densities were measured for TRAP^+^ osteoclasts and cuboidal osteoblasts lining the trabecular surface in the secondary spongiosa (cells/mm^2^ trabecular area), as well as adipocytes in the lower secondary spongiosa (cells/mm^2^ bone marrow area) as we described [23,25].

### 4.3. Serum Alkaline Phosphotase (ALP) Concentration

To investigate the effects of MTX, genistein, and the combination (Gen/MTX) treatments on bone formation, serum levels of alkaline phosphatase (ALP, a marker of osteoblast differentiation and activity) were measured using Konelav 20XT clinical chemistry analyzer (Thermo Scientific, Vantaa, Finland) as outlined previously [63].

### 4.4. Ex Vivo Osteoclastogenic Formation

*Ex vivo* osteoclast formation was performed as an indicator of treatment effects on the potential of the bone marrow precursor cells to form mature osteoclasts. As performed previously [25], whole bone marrow flushed from both femurs, the remaining tibia, and both humeri was combined and cultured overnight in basal media containing alpha-minimal essential medium (α-MEM) (Sigma, Sydney, Australia), 10% fetal calf serum (FCS) (Invitrogen, Carlsbad, CA, USA), 15 mM 4-(2-hydroxyethyl)-1-piperazineethanesulfonic acid (HEPES) (Sigma), 50 μg/mL Pen/Strep (Invitrogen), and 130 μM l-ascorbate-2-phosphate (Sigma). At the following day, the non-adherent bone marrow mononuclear cells (BMMNCs) were isolated and seeded at 1 × 10^6^ cells/well into 48-well plates in triplicate/treatment and supplemented with macrophage colony stimulating factor (M-CSF) (Peprotech, Rocky Hill, NJ, USA). After 24 h, osteoclastogenesis was induced over the following seven days with the addition of receptor activator of NF-κB ligand (RANKL) (Peprotech, Rocky Hill, NJ, USA) at 30 ng/mL to the culture medium. Osteoclast cultures were then formalin-fixed and stained for TRAP. Osteoclasts were then viewed under a light microscope and quantified as TRAP^+^ multinucleated osteoclasts (≥3 nuclei)/mm^2^ as described [25].

### 4.5. RNA Isolation and RT-PCR Gene Expression Analyses

Levels of mRNA expression of osteoclastogenesis, osteoblastogenesis, and adipogenesis-related genes were examined by RT-PCR using RNA isolated from frozen metaphysis samples, including RANKL and OPG, pro-inflammatory cytokines TNF-α, IL-1, and IL-6, osteoblast activity marker osteocalcin (OCN) and adipocyte differentiation factor C/EBP-α [24,29]. Using 2 μg RNA, cDNA was synthesised using Superscript III (Applied Biosystems, Sydney, Australia). RT-PCR was subsequently performed using an Applied Biosystems 7500 Fast Real-Time PCR system (Applied Biosystems, Sydney, Australia). Reactions of 20 μL were performed using 100 ng/mL cDNA, and amplification of product was measured via SYBR green fluorescence relative to endogenous cyclophilin (CYC) and expressed in the comparative *C*t format (2^−Δ*C*t^). Primer sequences for each gene of interest are provided in Table 1 (all primers ordered from Geneworks, Adelaide, Australia).

**Table 1:** Forward and reverse primer sequences used for mRNA expression analysis. Cyclophilin A (Cyc A), receptor activator of NF-κB ligand (RANKL), osteoprotogerin (OPG), tumor necrosis factor-alpha (TNF-α), interleukin-1 beta (IL-1β), interleukin-6 (IL-6), and CCAAT/enhanced binding protein-α (C/EBPα).

Gene	Forward Primer (5′–3′)	Reverse Primer (5′–3′)
*Cyc A*	CGTTGGATGGCACGCCTGTG	TGCTGGTCTTGCCATTCCTG
*RANKL*	TGGGCCAAGATCTCTAACATCAC	TGGGCCAAGATCTCTAACATCAC
*OPG*	AGCTGGCACACGAGTGATGAA	CACATTCGCACACTCGGTTGT
*TNF-α*	TGGGCCAAGATCTCTAACATCAC	CTCCGCTTGGTGGTTTGCTACGAC
*IL-1β*	GTTTCCCTCCCTGCCTCTGACA	GACAATGCTGCCTCGTGACC
*IL-6*	GATACCCACAACAGACCAG	GCCATTGCACAACTCTTTTCTC
*C/EBPα*	TCGCCATGCCGGGAGAACTCTAAC	CTGGAGGTGGCTGCTCATCGGGG

### 4.6. Statistical Analysis

All statistics analyses were performed using a standard one-way ANOVA with Tukey’s *post hoc* test (using GraphPad Prism 5.01 for Windows, GraphPad Software, San Diego, CA, USA) and data are represented as mean ± standard error of the mean (SEM). Significance was set at *p* < 0.05, where significance compared to control group is indicated in the graphs as *, **, and *** representing *p* < 0.05, *p* < 0.01, and *p* < 0.001 respectively, and differences between other treatment groups are exhibited by symbols “δ” or “#” with significance explained in respective figure legends.

## 5. Conclusions

Whilst genistein was found to have a limited ability to significantly protect against MTX-induced bone damage in some parameters, genistein supplementation suppressed MTX-induced osteoclast formation and showed protective effects on the general health and growth of the MTX-treated animals in this acute MTX treatment setting. Future studies are warranted to explore its potential efficacy in attenuating MTX chemotherapy-induced bone and bone marrow damages.
